# Applications of indocyanine green in robotic urology

**DOI:** 10.1007/s11701-016-0641-5

**Published:** 2016-09-23

**Authors:** Anthony S. Bates, Vipul R. Patel

**Affiliations:** 1 Department of Urology, University Hospitals of Leicester, Leicester, UK; 2 University of Oxford, Oxford, UK; 3 Global Robotics Institute, Florida Hospital in Celebration, Orlando, FL USA

**Keywords:** Indocyanine green, Robotic urology, RAPN, RARP, Intraoperative imaging, ICG, Da vinci

## Abstract

Indocyanine green is a fluorescent molecule with wide ranging applications in minimally invasive urological surgery. This article explores the utility of ICG assisted intraoperative fluorescence in robotic urology.

Indocyanine green (ICG) is a fluorescent molecule [1]. Incident infrared light of wavelength 780 nm provokes detectable photon emission at 820–830 nm [1]. Alongside a high definition camera and software imposed pseudo-colour, intravenously delivered ICG may be used to identify vessel perfusion and differentiate tissue density [2–4]. Indocyanine green was initially developed in the 1955 by Kodak photography, and received FDA approval in 1959 [5, 6]. Indocyanine green has a favourable safety profile, with an adverse event rate of 0.34 % including nausea, vomiting, and rarely shock [1 in 300,000]) [7]. The use of ICG is established in ophthalmology, dermatology, and cardiology for vascular identification [8, 9]. This article will summarise the current and future applications of ICG in robotic urology, with the da Vinci^®^ robot (dVSS), (Intuitive Surgical Systems, Sunnyvale, CA, USA) with emphasis placed on the intraoperative identification of vascular and oncological tissue. Notably, ICG is a cheap consumable, and infrared endoscopic equipment is widely available in contemporary dVSS systems (Firefly^®^ in the DaVinci Xi).

A historical perspective of intraoperative imaging with fluorescent dye is necessary to describe intra-operative imaging advances from the 20th century applicable to modern robotic surgery. In 1947, seminal work was conducted by neurosurgeon George Moore at the University of Minnesota Medical School. Moore described the use of fluorescein, a fluorophore, to identify intracranial neoplastic tissue in the journal *Science* [10]. By 1948, Moore had performed a case series of 46 patients with mixed intracranial tumours [11]. Intraoperatively, he injected tumour with dye, identifying 44 tumours (96 %) correctly as malignant [11]. Moore went so far as to describe resection of remaining tissue based on fluorescent activity, thereby intraoperatively resecting positive surgical margins [11]. He also radiolabelled diiodofluorescein with iodine isotope 131 to detect gamma radiation from the accumulation of dye at malignant tissue [11].

Complex pelvic surgery has been revolutionised by the dVSS [12]. Wide adoption of the dVSS for radical prostatectomy and partial nephrectomy has followed, due to enhanced degrees of movement from the robotic wrist, tremor reduction, enhanced ergonomics, greater magnification, and the ability to operate in a closed anatomical space. Improved surgical recovery has been realised due to the minimal access approach, reductions in blood loss and a favourable learning curve, compared with the conventional laparoscopic technique [13–15]. Robotic outcomes are comparable to laparoscopic approaches and superior to open techniques [13–15]. Clinician and patient preference for use of the dVSS in performing robotic partial nephrectomy and prostatectomy are reflected clearly in the increasing caseload undertaken using the platform, with hundreds of centres now offering robotic prostatectomy worldwide [16]. Despite advances in robotics such as virtual reality simulation and adaptations to instruments, the basic configuration of the dVSS has remained relativley unchanged. Image guided surgery is well established in urology, such as x-ray guided fluoroscopic investigation of the urethra, bladder, ureter and reno-ureteric junction [17]. The combination of fluorescent intraoperative imaging and robotic surgery is a logical progression in peri-operative tissue visualisation. The fusion of minimally invasive surgery and localised imaging has been described in the identification of sentinel lymph nodes and renal cancer [18, 19], super selective arterial clamping for nephron sparing [20], and precise dissection of the prostatic neurovascular bundle [21].

In healthy renal parenchyma the transporter bilitranslocase binds ICG and healthy tissue appears isofluorescent when perfused with ICG laden blood [22]. Renal tumour is deficient in bilitranslocase and therefore appears hypofluorescent [19]. Tobis et al. noted hypofluorescent renal tumours in the presence of ICG during robotic partial nephrectomy (RAPN). The investigators were guided by ICG in selective arterial clamping of a handful of cases. Following this, Manny et al. subsequently calculated hypofluorescent tissue had a sensitivity of 84 % and positive predictive value of 87 % for malignant renal lesions in 100 RAPN cases [23]. Borofsky et al. then described super selective renal artery clamping in 27 patients undergoing partial nephrectomy, with ICG, thus avoiding clamps to healthy renal parenchyma. Maximal loss of eGFR at 3 month follow-up was 1.6 versus 14.0 % in the non-ICG study arm [24]. Bjurlin et al. also reported a comparatively favourable 6.2 % decrease in glomerular filtration rate at 2 weeks postoperatively through the use of super-selective arterial clamping assisted with ICG [20, 25]. Patients undergoing super selective clamping showed significantly improved renal function at follow-up, however, these studies were underpowered and did not examine the impact of the technology upon intraoperative decision making [25].

Robot-assisted radical prostatectomy (RARP) may be supplemented by ICG imaging to identify the prostatic neurovascular bundle. In 2015, Patel et al. at demonstrated 30 % of prostatic neurovascular dissections were revised during nerve sparing RARP [21]. Given the degree of nerve spare correlates to functional outcomes [26], ICG holds promise for improving post-prostatectomy continence and erectile function. Robot-assisted sentinel node harvest is performed at RARP to determine metastatic nodal status. A hybrid fluorescent ICG radiotracer was optimised to detect sentinel lymph nodes in robot-assisted lymph node dissection, and improved in vivo identification through fluorescence to 93.5 % versus 50.0 % in non-optimised samples (*n* = 38; *p* = 0.005) [18]. In another series, robotic ICG assisted sentinel node harvest during RARP yielded a sensitivity of 100 % and negative predictive value of 100 % (*n* = 50), however, this method was non-specific. With larger sample sizes, it is likely the diagnostic coefficients would fall to below 100 % [27]. In spite of this, the potential utility of ICG for metastatic node detection and differentiation of oncological tissue remains encouraging.

Early work by Moore in revising surgical margins based on fluorescence, and the radiolabelling of fluorescent dye to localise oncological tissue was visionary. Recent technological advances in cancer biomarkers and immunology have prompted the hybridisation of ICG with cancer selective ligands, to localise tumour by fluorescence. Prostate specific membrane antigen (PSMA) is upregulated in prostate cancer by 100 to 1000-fold [28, 29]. Although at the pre-clinical stage, ICG bound PSMA-ligand was demonstrated to detect PSMA positive prostatic tumours in mice by Nakajima et al. using the humanised monoclonal antibody specific to PSMA, J591 [30]. Notably the J591 antibody has been delivered in several human clinical trials at high doses with a favourable safety profile [31–33]. In mice with human cell line prostate cancer, tumour was visually identifiable through fluorescence from 1 to 10 days after administration [30]. The accurate identification of previously indiscernible cancer tissue should be expected to transform positive surgical margin rates, and delay biochemical recurrence in prostate cancer. Similar advances in renal and bladder cancer immunology may also allow transfer of the technology to treat these pathologies.

In summary ICG combined with the dVSS is an incumbent game changer, and has been described as a “hammer looking for a nail” [25]. Evidence is emerging that ICG is useful in the robotic surgeon’s arsenal and yields tangible clinical benefit from selective arterial clamping in RAPN. Well-designed randomised controlled trials are urgently needed to quantify improvements in outcomes following ICG-augmented robotic procedures such as sentinel node excision, and identification of oncological tissue. It remains to be proven whether ICG brings small, incremental procedure-specific advances, or clinically substantial benefits to overall survival and functional recovery to these procedures. A revolutionary step in operative imaging though the labelling of fluorescent molecules to enhance en bloc tumour resection was espoused nearly 70 years ago by neurosurgeon Dr George Moore. Although at the pre-clinical stage, it is only a matter of time before Moore’s pioneering work is assimilated into routine robotic surgical practice.
